# The influence of benevolent leadership on knowledge sharing of postgraduate supervisor: A moderated mediating model

**DOI:** 10.3389/fpsyg.2022.1071442

**Published:** 2022-12-08

**Authors:** Xiaoyu Li, Dongdong Gao

**Affiliations:** ^1^School of Philosophy and Public Administration, Henan University, Kaifeng, China; ^2^Institute of Psychology and Behavior, Henan University, Kaifeng, China

**Keywords:** benevolent leadership, knowledge sharing, creative self-efficacy, professional identity, research stress

## Abstract

In order to explore the mechanism and boundary conditions of the influence of benevolent leadership on knowledge sharing, we took postgraduate supervisor as participants and constructed a moderated mediating effect model. In this study, a total of 1,083 valid questionnaires were collected by questionnaire method and the confirmatory factor analysis, correlation analysis, regression analysis, and Hayes’s PROCESS macro were used to analyze the data. The results show that benevolent leadership positively affects knowledge sharing. Creative self-efficacy mediates the relationship between benevolent leadership and knowledge sharing. Professional identity moderates the relationship between benevolent leadership and creative self-efficacy, when the professional identity is (M − 1 SD) and (M + 1 SD), the moderating effect is significant, while when the professional identity is (M), the moderating effect is not significant. Research stress moderates the relationship between creative self-efficacy and knowledge sharing, when research stress is (M − 1 SD), (M), and (M + 1 SD), the moderating effect is significant. Professional identity and research stress jointly moderated the mediating effect of creative self-efficacy. Professional identity moderated the first half path of the mediating model, while research stress moderated the second half path of the mediating model. When the level of professional identity is high and research stress is high, benevolent leadership has the greatest positive influence on knowledge sharing through creative self-efficacy. When the level of professional identity is low and research stress is high, benevolent leadership has the greatest negative influence on knowledge sharing through creative self-efficacy. This study enriches the relevant research on benevolent leadership and knowledge sharing, explores the conditions and factors that enhance or buffer benevolent leadership, and shows that the best effect can be achieved when the leadership behavior is consistent with the situational factors.

## Introduction

With the development of economy and society, the competition among universities is becoming more and more fierce, and knowledge sharing plays an increasingly important role in winning competitive advantages for universities. Postgraduate supervisors whose main task is talent cultivation and scientific research play an important role in knowledge inheritance, innovation and sharing (Lu, 2017). What methods the leaders take and what conditions they create to encourage postgraduate supervisors to share knowledge, so as to improve the quality of talent cultivation and scientific research to enhance the competitiveness of schools, has become a problem that all universities must solve. However, in the existing literature, there are few studies on knowledge sharing of postgraduate supervisors. The purpose of this study is to explore what factors influence the knowledge sharing of postgraduate supervisors, and what are the action mechanism and boundary conditions of this influence.

In the organization, not everyone is willing to share knowledge (Sedighi et al., 2016), the main reason is that individuals have the risk of being surpassed and replaced by others after knowledge sharing (Su et al., 2018, 2021, 2022). The factors that affect knowledge sharing can be divided into organizational factors and individual factors (Li et al., 2014; Xu et al., 2020). Among organizational factors, the influence of leadership on knowledge sharing deserves attention. Leaders can influence knowledge sharing not only through their own behaviors, but also through organizational culture, atmosphere, values, and institutional system (Le and Lei, 2018; Le and Than, 2020; Than et al., 2020). Benevolent leadership provide comprehensive and personalized compassion and care for the work and life of their subordinates (Cheng et al., 2002), according to the social exchange theory (Blau, 1964), subordinates will reciprocate with corresponding work achievements or altruistic behaviors (Farh et al., 2006). For postgraduate supervisors, knowledge sharing is a practical and efficient way to pay back when they reward benevolent leaders. It can be concluded that benevolent leadership has a positive role in promoting the knowledge sharing behavior of postgraduate supervisors.

Among the individual factors affecting knowledge sharing, self-efficacy is an important factor (Liu and Liu, 2011). The creative self-efficacy is the embodiment of self-efficacy in the field of creation (Tierney and Farmer, 2002, 2011), the employees with a high sense of creative self-efficacy have a stronger desire to share knowledge (Yoon and Han, 2018). Knowledge sharing is largely limited by individual abilities and qualities. When postgraduate supervisors are confident in their knowledge, ability, and creative thinking, they will have a higher sense of creative self-efficacy and a higher willingness to share knowledge, and vice versa. Individual creative self-efficacy is influenced by leadership behavior. Benevolent leadership not only understands postgraduate supervisors, help them solve their problems, but also creates a relaxed working environment. Benevolent leadership promotes the improvement of postgraduate supervisors’ creative self-efficacy. Therefore, it can be inferred that creative self-efficacy plays a mediating role between benevolent leadership and knowledge sharing.

Although benevolent leadership can positively affect creative self-efficacy, this relationship is affected by the attitude of individuals to the work they are engaged in. If individuals have a high degree of identification with the work they are engaged in, the positive influence of benevolent leadership on creative self-efficacy will be enhanced. If individuals have a low degree of identification with the work they are engaged in, the negative influence of benevolent leadership on creative self-efficacy will be enhanced (Wu et al., 2012; Li et al., 2018). The relationship between creative self-efficacy and knowledge sharing will also be affected by external conditions. Combines the actual conditions of the work of postgraduate supervisors, this manuscript considers research stress as an important environmental factor. Because research stress is a challenging stress, it can be considered that the relationship between creative self-efficacy and knowledge sharing is closer when research stress is high.

**FIGURE 1:**
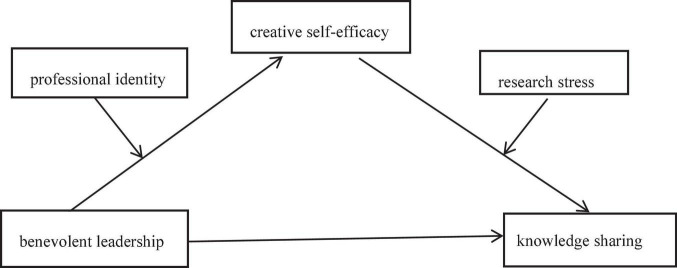
Hypothesized model.

The influence of different leadership styles on knowledge sharing has been studied in the previous literature, the differences between this study and previous studies are as follows: First, take the postgraduate supervisors as the participant for research. Second, benevolent leadership is studied as a separate variable from the content of paternalistic leadership. Third, design two moderating variables, not only to examine the role of each moderating variable, but also to explore the jointly role of the two moderating variables. This study focuses on the following questions: the influence of benevolent leadership on knowledge sharing of postgraduate supervisors; the mediating effect of creative self-efficacy; the moderating effect of professional identity and research stress; how professional identity and research stress jointly moderate the mediating effect of creative self-efficacy between benevolent leadership and knowledge sharing. The specific theoretical model is shown in Figure 1.

## Theory and hypotheses

### The influence of benevolent leadership on knowledge sharing

Benevolent leadership refers to the individual, comprehensive, and long-term care shown by leaders for the personal well-being of their subordinates (Cheng et al., 2004). The benevolent of leaders refers to the individual care shown by leaders to their subordinates. This kind of benevolent is not distributed to all subordinates equally, but varies according to the contributions and interests of subordinates (Farh and Cheng, 2000). The idea that human nature is good and people-oriented is the philosophical basis for the generation of benevolent leadership. Benevolent leadership leads others through virtue and emphasizes the interaction of social relations. When all parties can fulfill their roles dutifully, they can maintain the harmony of relations (Zhang et al., 2013b). Although the dimension of personalized care in transformational leadership is similar to that of benevolent leadership, the difference between them is still relatively large. Transformational leadership originates from the Western cultural background. The meaning of the dimension of personalized care mainly refers to the concern of leaders for the problems of subordinates in work, providing guidance, support, and help for subordinates in work, but rarely involving the problems of subordinates in personal life. However, while caring about the work level of their subordinates, benevolent leadership also extend it to the life level. In particular, when their subordinates’ families encounter difficulties in life, leaders will generally give assistance within their capabilities, such as helping their families to seek medical treatment and helping their children to go to school (Li et al., 2016). Benevolent leaders’ concern and help for their subordinates in their work and life will inevitably promote their subordinates to have positive behaviors and positive returns. Previous studies have shown that benevolent leadership has a significant positive predictive effect on work engagement (Xu et al., 2018), altruistic behavior (Gumusluoglu et al., 2019), work performance (Jin et al., 2016), psychological empowerment (Chan, 2017), psychological well-being (Erkutlu and Chafra, 2016), creativity behavior (Lin et al., 2018).

Knowledge sharing is that individuals provide task information and experience to help others, through the process of working with others to solve problems, develop new ideas and implement new processes (Ipe, 2003). In essence, knowledge sharing is a process in which individuals exchange knowledge and create new knowledge together (Wang and Noe, 2010). Knowledge sharing is a voluntary behavior of individuals. It is a process in which knowledge owners voluntarily transfer knowledge to others so that others can use the knowledge (Saeed et al., 2022). Even if there is no such requirement in the rules and regulations of the unit, knowledge owners are obligated to transfer knowledge (Su et al., 2022). Since knowledge sharing can make the information of individuals with different majors, backgrounds, experiences, knowledge, and skills in the organization gather together, and then break the barriers between knowledge owners and realize the free flow of knowledge within a certain range, knowledge sharing has a very significant positive effect on the organization (Wang et al., 2012; Bavik et al., 2018; Jiang and Chen, 2021). Knowledge sharing has a “double-edged sword” effect on individuals. On the one hand, in the process of knowledge sharing, individual knowledge is reorganized and integrated, and new ideas and ideas are generated in the process of sharing with others (Dong et al., 2017). Knowledge sharing plays the role of “individual gain.” On the other hand, in the process of knowledge sharing, after others master the relevant knowledge, the individual’s knowledge advantage will be lost, leading to the weakening of competitiveness (Su et al., 2018, 2021, 2022). Knowledge sharing plays the role of “individual loss.” Therefore, how to motivate employees to share knowledge has become a problem that organizations must face (Wu and Lee, 2017; Wu, 2021).

Multiple studies have demonstrated that leadership can influence knowledge sharing. Empowering leadership promote knowledge sharing by letting subordinates take responsibility, participate in decision-making, and communicate with each other (Srivastava et al., 2006). Transformational leadership promote knowledge sharing by building common goals, reducing self-interest motives, and providing personalized care (Shih et al., 2012; Liu and DeFrank, 2013; Dong et al., 2017). Self-sacrificial leadership promote knowledge sharing by emphasizing the mission and objectives of the organization and setting an example (Su et al., 2022). Benevolent leaders will not only give more attention and care to postgraduate supervisors in work, but also take care of many things outside of work or family members. According to the “reciprocity rule” in the social exchange theory (Blau, 1964), when one party receives favors and help from the other party, it will actively take actions to give the other party the same value in return (Gao and Liu, 2021). Benevolent leadership’s psychological and material support behavior will make the postgraduate supervisors feel grateful and return. This return is not only limited to the leader himself, but also spills over to the organizational level. For postgraduate supervisors, knowledge sharing is the most effective and convenient way for them to repay their leaders and organizations. Therefore, it is inferred that benevolent leadership positively predicts knowledge sharing behavior of postgraduate supervisors. Based on this, hypothesis 1 is proposed:

Hypothesis 1: Benevolent leadership can positively predict knowledge sharing of postgraduate supervisors.

### The mediating effect of creative self-efficacy on the relationship between benevolent leadership and knowledge sharing

Creative self-efficacy is an individual’s evaluation of whether a certain work he or she is engaged in has the ability and confidence to produce creative behavior, which reflects the individual’s self-belief or expectation for himself or herself in creative activities (Tierney and Farmer, 2002). Yang et al. (2011) further explained the creative self-efficacy as the special self-efficacy of individuals in their creative activities and the expression of their belief in the realization of their creativity. It can be understood from four aspects: the belief that you can generate new ideas, the confidence that you can solve problems creatively, the skill and impulse to help others complete new ideas, and the confidence that you can find solutions to new problems (Yang et al., 2011). Previous studies have shown that creative self-efficacy has a significant positive predictive effect on innovation behavior (Mielniczuk and Laguna, 2018; Newman et al., 2018), creative performance (Tierney and Farmer, 2011), and is an important factor to promote organizational development (Puente-Diaz, 2016). The influencing factors of creative self-efficacy can be roughly divided into organizational factors, leadership factors, job-related factors, individual factors, etc. (Yang and Cheng, 2009; Yang et al., 2011). Among them, the positive influence of leadership factors on creative self-efficacy has been supported by many studies (Gong et al., 2009; Gu et al., 2015; Ye et al., 2018, 2019).

Benevolent leaders’ personalized care and compassion for subordinates and their families in work and life can improve the creative self-efficacy of postgraduate supervisors in the following two aspects. First, the encouragement and support of benevolent leaders to the work of their subordinates provides a safe psychological environment, stimulates the role obligation of postgraduate supervisors, increases work confidence, and enables postgraduate supervisors to dare to generate and try new ideas without worrying about the negative impact of failure (Emilia and Mariola, 2018). Secondly, benevolent leader’s concern for the personal life and family of subordinates can make the postgraduate supervisor concentrate more time and energy on the work, and avoid trifles to delay and affect the work (Chen et al., 2014). Therefore, the postgraduate supervisor can concentrate on creative work, and thus improve the personal sense of creative self-efficacy. The positive effect of benevolent leadership on creative self-efficacy has been proved by relevant studies (Xia et al., 2022).

Creative self-efficacy is the specific application of individual self-efficacy in creative activities, is the subjective evaluation of individual’s creative ability, and is the premise and basis of creative behavior. Postgraduate supervisors with high creative self-efficacy tend to be more willing to share knowledge. There are two main reasons. First, knowledge sharing helps postgraduate supervisors improve their expert power and organizational status (Wipawayangkool and Teng, 2016). Postgraduate supervisors with a high creative self-efficacy are more confident. They believe that they can generate new ideas in their study and work, like to think and explore work with new eyes and perspectives, and also believe that they can help others solve problems or promote the development of the organization through their own knowledge sharing (Yoon and Han, 2018; Wang et al., 2020). Therefore, they are more likely to be respected by others and valued by the organization. Second, knowledge sharing is conducive to the improvement of the quality of postgraduate supervisors. The knowledge sharing process of postgraduate supervisors is not only a process for others to understand and accept knowledge, but also a process in which individual knowledge becomes clearer, organized, systematic and scientific. In the process of sharing knowledge, individuals must first think, sort out, summarize their own knowledge before sharing it, so in the process of knowledge sharing, postgraduate supervisors will also have greater benefits (Tang et al., 2020; Tantawy et al., 2021).

According to the social exchange theory (Blau, 1964), caring, support, tolerance, and other behaviors of benevolent leadership have a positive influence on the creative self-efficacy (Yang et al., 2011). According to the content related to self-efficacy in social cognitive theory (Bandura, 1977), it can be inferred that the creative self-efficacy has a positive influence on knowledge sharing (Xu et al., 2021). Therefore, it can be concluded that the benevolent leadership promotes the knowledge sharing of the postgraduate supervisors by improving the creative self-efficacy. Based on this, hypothesis 2 is proposed:

Hypothesis 2: Creative self-efficacy mediates the relationship between benevolent leadership and knowledge sharing.

### The moderating effect of professional identity on the relationship between benevolent leadership and creative self-efficacy

Professional identity is an individual’s positive attitude and strong sense of investment in a certain occupation, which is reflected in the desire of the individual to maintain the occupation and the degree of love for the occupation (Lasky, 2005; Tripathi et al., 2020). Professional identity is based on the theory of social identity (Zhao et al., 2022), influenced by individual characteristics, the cultural atmosphere at that time and the evaluation of a certain career by social groups, and according to individual characteristics and social evaluation to determine their feelings and value significance of the occupation (Sheybani and Miri, 2019; Wei, 2021). Teachers’ professional identity is the synthesis of teachers’ positive cognition, experience, and behavioral tendency toward their profession and their internalized professional role (Mahmoudi-Gahrouei et al., 2016; Sardabi et al., 2018). It is a multi-dimensional structure composed of four factors: professional value, role value, professional sense of belonging, and professional behavior tendency (Wei et al., 2013). Previous studies have shown that teachers’ professional identity, as a protective factor, plays an enhanced role in job engagement, job satisfaction, career happiness, career commitment, professional development, job performance, mental health, and other factors (Ng and Feldman, 2008; Hong, 2010), while reducing occupational stress, job burnout, turnover intention, and other negative factors (Beijaard et al., 2000; Wei et al., 2013).

Professional identity can be divided into different levels such as high and low, strong and weak, so it is used as a moderating variable in this study. Individuals with high professional identity have a positive attitude toward work (Beijaard et al., 2004), pay attention to the accumulation of professional knowledge and the improvement of professional skills (Wang et al., 2011), are willing to pay time and energy for work, and aspire to achieve work achievements, and have a good career development prospect (Luo et al., 2014). Individuals with low professional identity have a negative attitude toward work, are not willing to pay in the work, are prone to job burnout and negative emotions (Zhao et al., 2022), and are prone to turnover when encountering setbacks (Chen et al., 2020). In this study, professional identity as an individual variable moderates the relationship between benevolent leadership and creative self-efficacy. When postgraduate supervisors have a high level of professional identity, the relationship between benevolent leadership and creative self-efficacy is positively enhanced. Because when postgraduate supervisors have a positive attitude toward the work, the benevolent leadership’s care, support, encouragement, and other behaviors strengthen the creative self-efficacy of postgraduate supervisors (Yang et al., 2011). When postgraduate supervisors have a low level of professional identity, the relationship between benevolent leadership and creative self-efficacy is negatively enhanced. The main reason for this situation is that when the individual’s professional identity is low, he will have a negative attitude toward work, and his requirements for his work will continue to decrease (Ding et al., 2022). The kindness and support of benevolent leadership, and even the tolerance of “those who make mistakes,” are regarded as “lax requirements,” “acquiescence in deviant behavior,” and “weak and incompetence,” which leads to worse and worse results (Li et al., 2022). Therefore, hypothesis 3 is proposed:

Hypothesis 3: Professional identity plays a moderating role between benevolent leadership and creative self-efficacy. The higher the professional identity, the stronger the positive influence of benevolent leadership on creative self-efficacy. The lower the professional identity, the stronger the negative influence of benevolent leadership on creative self-efficacy.

### The moderating effect of research stress on the relationship between creative self-efficacy and knowledge sharing

Research stress refers to the stress generated in the process of completing scientific research tasks or carrying out scientific research activities. Stress has both positive and negative effects. Depending on the different effects, it can be divided into enstress and distress (Selye, 1982). According to the nature of work stress and its effects, the challenge-hindrance model of stress divides stress into challenge stressors and hindrance stressors (Cavanaugh et al., 2000). Challenge stressors is a kind of stress that can promote the growth and development of individuals (Rodell and Judge, 2009). It is mainly related to workload, time pressure, job complexity, and job responsibilities (Crawford et al., 2010). Hindrance stressors is a kind of stress that hinders the development of individual ability and work harvest (Boswell et al., 2004; LePine et al., 2005). It is mainly related to job insecurity, role conflict, and work distress (Podsakoff et al., 2007; Mazzola and Disselhorst, 2019). For postgraduate supervisors, the research stress should belong to the challenge stress, because scientific research is one of the main work tasks of postgraduate supervisors, and many of the assessment indicators of postgraduate supervisors are related to scientific research. Research stress is something that every postgraduate supervisor will encounter in their work.

As an environmental variable, research stress plays a moderating role in the relationship between creative self-efficacy and knowledge sharing of postgraduate supervisors. First, under the condition of high research stress, it arousal the postgraduate supervisors’ research needs and motives, and has an incentive effect on research behavior (Bunce and West, 1994). Postgraduate supervisor adopt a positive attitude and behavior to cope with the stress. Knowledge sharing is conducive to the integration and innovation of personal knowledge and the completion of scientific research tasks. Therefore, the higher the research stress, the closer the relationship between creative self-efficacy and knowledge sharing. Second, the affective events theory regard (Weiss and Cropanzano, 1996), affective reactions and work behaviors of different individuals are different for the same stressful event (Duan et al., 2011). The transactional theory of stress regard (Lazarus and Folkman, 1984), the influence of stress on individual psychology and behavior depends on individual evaluation and judgment of stress. According to the above two theories, under the condition of high research stress, postgraduate supervisors will regard research stress as challenges and opportunities, and they will take positive behaviors to cope with the stress, so they will have more knowledge sharing behaviors. While under the condition of low research stress, due to insufficient stress and other reasons, the enthusiasm of postgraduate supervisors to share knowledge is also low. Based on this, hypothesis 4 is proposed:

Hypothesis 4: Research stress plays a moderating role between creative self-efficacy and knowledge sharing. The greater the research stress, the closer the relationship between creative self-efficacy and knowledge sharing will be.

### A moderated mediating effect among the five variables

According to the above explanation, this study believes that benevolent leadership can be used as the independent variable, knowledge sharing as the dependent variable, creative self-efficacy as the mediating variable, and professional identity and research stress as the moderating variable to form a moderated mediating effect model. Due to the complexity of the situation, the mediating effect of benevolent leadership on knowledge sharing through creative self-efficacy is likely to be affected by more than one moderating variable. In this study, professional identity as an individual variable and research stress as an environmental variable jointly moderated the mediating effect of benevolent leadership on knowledge sharing through creative self-efficacy. Professional identity moderated the first half of the mediating effect model, while research stress moderated the second half of the mediating effect model.

The “motivation-opportunity-ability framework” of individual behavior believe (Enno et al., 2008), a behavior is most likely to occur when the motivation, ability, and opportunity are combined (Blumberg and Pringle, 1982; Abiero and Bradfield, 2021). The effectiveness of this framework has been verified in many fields. In the field of knowledge management, it is mainly used to explain knowledge transmission, knowledge exchange, and knowledge sharing (Tobin and Wesley, 2015; Elbaz et al., 2018). This framework can also be used in this study to explain the influence of independent variables, mediating variables and moderating variables on knowledge sharing. Benevolent leadership as a “motivation factor” influence knowledge sharing, creative self-efficacy as a “ability factor” influence knowledge sharing, professional identity, and research stress together as a “opportunity factor” influence knowledge sharing. As the moderating effect is jointly acted by professional identity and research stress, it can be divided into four types: high professional identity and high research stress, high professional identity and low research stress, low professional identity and high research stress, low professional identity and low research stress. Since professional identity reflects an individual’s attitude toward work and research stress reflects the challenge stress felt by an individual. We infer that when an individual’s professional identity is high and research stress is high, both of them have a positive moderating effect on the mediating effect of creative self-efficacy; when an individual’s professional identity is low and research stress is high, both of them have a negative moderating effect on the mediating effect of creative self-efficacy. Accordingly, hypothesis 5 is put forward as follows:

Hypothesis 5: Professional identity and research stress jointly moderate the mediating effect of benevolent leadership on knowledge sharing through creative self-efficacy. The higher the professional identity and research stress, the stronger the positive moderating effect is on the mediating effect of creative self-efficacy, while the lower the professional identity and the higher research stress, the stronger the negative moderating effect is on the mediating effect of creative self-efficacy.

## Materials and methods

### Procedure and participants

This study collects data through questionnaire survey. Using personal social network relationship and snowballing method, the questionnaire was distributed to many universities in China. The online survey and on-site survey were used to collect the questionnaire. The online survey was carried out through professional platform what is named “Wenjuanxing,” the on-site survey was carried out by sending questionnaires to the participants and taking them back after answering them. The confidentiality of the results was emphasized before the survey, and the questionnaires were filled out voluntarily by the participants. A total of 1,211 questionnaires were distributed, after collecting the questionnaire, review the answers, delete linear and wavy answers, and 1,083 valid questionnaires were collected. Table 1 presents the demographic characteristics of the sample. Of the 1,083 participants, included 709 (65.466%) males and 374 (34.534%) females. 30 (3.139%) participants were aged 30 years and below, 163 (15.051%) were aged 31–35 years, 355 (32.779%) were aged 36–40 years, 242 (22.345%) were aged 41–45 years, 149 (13.758%) were aged 46–50 years, 112 (10.342%) were aged 51–55 years, and 28 (2.586%) were aged 56 years and above. There were 177 (16.343%) participants with lecturer titles, 557 (51.431%) with associate professor titles, and 349 (32.226%) with professor titles. With regard to the years as a postgraduate supervisor, 421 (38.873%) participants were 3 years and below, 257 (23.730%) were 4–6 years, 165 (15.236%) were 7–9 years, and 240 (22.161%) were 10 years and above.

**TABLE 1:** Demographic information of sample.

Characteristics	Item	n	%
Gender	Male	709	65.466
	Female	374	34.534
Age	30 years and below	34	3.139
	31–35	163	15.051
	36–40	355	32.779
	41–45	242	22.345
	46–50	149	13.758
	51–55	112	10.342
	56 years and above	28	2.586
Professional titles	Lecturer	177	16.343
	Associate professor	557	51.431
	Professor	349	32.226
Years as a postgraduate supervisor	3 years and below	421	38.873
	4–6	257	23.730
	7–9	165	15.236
	10 years and above	240	22.161

*N* = 1,083.

### Measurements

The measurement scales used in this study were derived from existing literature and have been used several times in published academic articles, showing good reliability and validity. All the scale items were rated on a five-point Likert scale, with 1 implying “completely disagree” and 5 implying “completely agree.”

#### Benevolent leadership

Benevolent leadership was measured using the Benevolent Leadership Scale developed by Fu et al. (2012), this scale is adapted from the scale developed by Cheng et al. (2000), which consists of five items. Samples of these items are as follows: “The leader will care about my personal life” and “The leader’s care for me will extend to my family.” The Cronbach’s alpha for this scale was 0.939.

#### Knowledge sharing

Knowledge sharing was measured using the Knowledge Sharing Intention Scale developed by Wang and Zhu (2012), which consists of five items. Samples of these items are as follows: “I am willing to share my knowledge and experience with others” and “When participating in the discussion, I will provide my own opinions as much as possible.” The Cronbach’s alpha for this scale was 0.881.

#### Creative self-efficacy

Creative self-efficacy was measured using the Creative Self-Efficacy Scale developed by Tierney and Farmer (2002), which consists of four items. Samples of these items are as follows: “I think I’m good at putting forward new ideas” and “I have confidence in my ability to solve problems creatively.” The Cronbach’s alpha for this scale was 0.877.

#### Professional identity

Professional identity was measured using the Chinese University Teachers Professional Identity Questionnaire developed by Zhang et al. (2013a), which consists of six items. Samples of these items are as follows: “As a postgraduate supervisors, I often feel respected” and “I am proud to be a postgraduate supervisors.” The Cronbach’s alpha for this scale was 0.848.

#### Research stress

Research stress was measured using the Research Stress Questionnaire developed by Zhang et al. (2013c), which consists of three items. Samples of these items are as follows: “I am worried about how to complete the research task” and “I feel a lot of stress from my research work.” The Cronbach’s alpha for this scale was 0.828.

#### Control variables

According to previous research, the gender, age, professional title, years as a postgraduate supervisor were used as control variables (Chen et al., 2022; Xia et al., 2022).

### Data analyses

Data analyses were conducted using SPSS23.0 and AMOS23.0 (Wu, 2009, 2010), all comparisons were two-tailed, and *p*-values < 0.05were considered statistically significant. First, to establish the validity of the data. Confirmatory factor analysis is used in AMOS23.0 to evaluate the discriminant validity and common method bias between the five variables (Wu, 2009). Second, descriptive analyses were conducted. Descriptive the mean, standard deviation, and Pearson Correlation test was used to measure the correlation between the variables (Wu, 2010). Third, hypothesis test were conducted. Before testing the model, all variables were standardized. Explore the direct effect of the benevolent leadership on knowledge sharing by regression analysis; Model 4 of PROCESS was employed to test whether creative self-efficacy mediated the effect of the benevolent leadership on knowledge sharing; Model 1 of PROCESS was employed to test whether professional identity moderated the effect of the benevolent leadership on creative self-efficacy and research stress moderated the effect of creative self-efficacy on knowledge sharing; Model 21 of PROCESS was employed to test the moderated mediating effect model composed of 5 variables such as benevolent leadership, knowledge sharing, creative self-efficacy, professional identity, and research stress (Hayes, 2013, 2017). In addition, mediating effect and moderating effect analyses were tested using non-parametric bootstrapping methods, the 95% confidence interval produced by the bootstrapping procedure was examined and if zero was not included within the confidence interval, the effect was considered significant (Preacher and Hayes, 2004; Preacher et al., 2007; Hayes, 2009).

## Research results

### Common bias test and discriminant validity

In order to control the bias effect of common methods, the scales with good reliability and validity are used as the measuring tools. In the test process, the confidentiality of the results and the use of the results for academic research only were emphasized. The common method bias was evaluated by control unmeasured single method-factor approaches (Podsakoff et al., 2003; Zhou and Long, 2004). Adding the common method variance (CMV) in confirmatory factor analysis, Table 2 reveals that the fitting degree of the “Five-factor model + CMV” model was not significantly improved (△*RMSEA* = 0.008 < 0.05, △*SRMR* = 0.007 < 0.05, △*TLI* = 0.016 < 0.1, △ *CFI* = 0.019 < 0.1). Thus, there is no serious common method bias in this data (Wen et al., 2018).

**TABLE 2:** Discriminant validity and common method bias test results.

Model	χ*^2^*	*df*	χ*^2^/df*	*CFI*	*TLI*	*RMSEA*	*SRMR*
Single-factor model	9187.715	230	39.947	0.394	0.333	0.190	0.159
Two-factor model a	7345.057	229	32.074	0.518	0.468	0.169	0.191
Two-factor model b	5297.192	229	23.132	0.657	0.621	0.143	0.120
Three-factor model a	4311.398	227	18.993	0.724	0.692	0.129	0.120
Three-factor model b	4900.563	227	21.588	0.684	0.648	0.138	0.149
Four-factor model a	2266.432	224	10.118	0.862	0.844	0.092	0.077
Four-factor model b	3096.804	224	13.825	0.806	0.780	0.109	0.102
Five-factor model	1044.978	220	4.750	0.944	0.936	0.059	0.043
Five-factor model + CMV	747.937	197	3.797	0.963	0.952	0.051	0.030

Single-factor model = BL + KS + CSE + PI + RS; Two-factor model a = BL + KS + CSE, PI + RS; Two-factor model b = BL, KS + CSE + PI + RS; Three-factor model a = BL, KS + CSE, PI + RS; Three-factor model b = BL + KS, CSE, PI + RS; Four-factor model a = BL, KS, CSE, PI + RS; Four-factor model b = BL, KS + CSE, PI, RS; Five-factor model = BL, KS, CSE, PI, RS; “+” represents factor combination; BL, benevolent leadership; KS, knowledge sharing; CSE, creative self-efficacy; PI, professional identity; RS, research stress; CMV represents homologous variance.

To test the discriminant validity between the five variables, the goodness of fit of each competing factor model was compared by confirmatory factor analysis. The results in Table 2 show that the fitting indicators of the “Five-factor model” (χ*2/df* = 4.750, *RMSEA* = 0.059, *SRMR* = 0.043, *TLI* = 0.936, *CFI* = 0.944) basically meet the standard and are significantly better than other factor models (Hair et al., 2019), which indicates that the research variables have good discriminant validity.

### Descriptive statistics and correlation analysis

Means, standard deviations, and correlations of the variables used in the analysis are presented in Table 3. The results reveal that the benevolent leadership is significantly positively correlated with knowledge sharing (*r* = 0.228, *p* < 0.01), creative self-efficacy (*r* = 0.197, *p* < 0.01), professional identity (*r* = 0.383, *p* < 0.01), and negatively correlated with research stress (*r* = −0.007, *p* > 0.05). Knowledge sharing is significantly positively correlated with creative self-efficacy (*r* = 0.351, *p* < 0.01), professional identity (*r* = 0.334, *p* < 0.01), and negatively correlated with research stress (*r* = −0.031, *p* > 0.05). Creative self-efficacy is significantly positively correlated with professional identity (*r* = 0.564, *p* < 0.01) and negatively correlated with research stress (*r* = −0.050, *p* > 0.05). Professional identity is positively correlated with research stress (*r* = 0.035, *p* > 0.05).

**TABLE 3:** Mean, standard deviation, and correlation analysis results of each variable.

Variable	1	2	3	4	5	6	7	8	9
1. Gender	1								
2. Age	−0.067*	1							
3. Title	−0.107**	0.613**	1						
4. SA	−0.060*	0.658**	0.628**	1					
5. BL	0.039	−0.135**	−0.094**	−0.134**	1				
6. KS	0.056	0.007	0.056	0.043	0.228**	1			
7. CSE	–0.095	–0.045	0.088**	0.036	0.197**	0.351**	1		
8. PI	0.046	–0.059	0.014	–0.011	0.383**	0.334**	0.564**	1	
9. RS	0.005	–0.026	−0.069*	−0.064*	–0.007	–0.031	–0.050	0.035	1
M	1.350	41.720	2.160	2.210	3.094	4.138	3.936	3.870	3.475
SD	0.476	6.966	0.679	1.177	0.934	0.470	0.591	0.605	0.882

*p < 0.05, **p < 0.01; Gender: 1, male; 2, female; Title: 1, lecturer; 2, associate professor; 3, professor; SA, Supervisor age: 1, 3 years and below; 2, 4–6 years; 3, 10 years and above; BL, benevolent leadership; KS, knowledge sharing; CSE, creative self-efficacy; PI, professional identity; RS, research stress; M, mean; SD, standard deviation.

### Hypothesis testing

The input method was used for linear regression analysis in SPSS, and the results revealed that the benevolent leadership positively influences knowledge sharing (*Model 1*, *B* = 0.236, *SE* = 0.032, *p* < 0.001). Therefore, Hypothesis 1 was verified.

In SPSS, Process Macro Model 4 (Hayes, 2013, 2017) was used for mediating effect analysis. Creative self-efficacy is a mediating variable, benevolent leadership is an independent variable, and knowledge sharing is a dependent variable. It can be seen from Table 4, benevolent leadership can positively influences creative self-efficacy (*Model 2*, *B* = 0.202, *SE* = 0.027, *p* < 0.001), creative self-efficacy can positively influences knowledge sharing (*Model 3*, *B* = 0.323, *SE* = 0.019, *p* < 0.001), benevolent leadership can positively influences knowledge sharing (*Model 3*, *B* = 0.170, *SE* = 0.019, *p* < 0.001). The direct effect of the model is 0.174 (*SE* = 0.030, *CI* [0.177, 0.294]), and indirect effect is 0.065 (*BootSE* = 0.013, *BootCI* [0.041, 0.093]), the mediating effect accounts for 27.643% of the total effect. Because creative self-efficacy has a significant mediating effect between benevolent leadership and knowledge sharing, hypothesis 2 is verified.

**TABLE 4:** Regression analysis results of mediating and moderating effects.

Variables	Model 1	Model 2	Model 3	Model 4	Model 5
	KS	CSE	KS	CSE	KS
Constant	–0.250	0.624	–0.452	0.488	–0.387
Gender	0.116	−0.196**	0.179**	−0.227***	0.203**
Age	–0.006	−0.024***	0.002	−0.016**	0.001
Title	0.104	0.229***	0.031	0.174***	0.026
SA	0.051	0.057	0.032	0.024	0.017
BL	0.236***	0.202***	0.170***	–0.035	
CSE			0.323***		0.364***
PI				0.590***	
BL × PI				0.130***	
RS					–0.029
CSE × RS					0.062**
*R*	0.251	0.268	0.400	0.607	0.372
*R* ^2^	0.063	0.072	0.160	0.368	0.138
*F*	14.514***	16.714***	34.081***	89.604***	24.643***

**p < 0.01, ***p < 0.001; SA, supervisor age; BL, benevolent leadership; KS, knowledge sharing; CSE, creative self-efficacy; PI, professional identity; RS, research stress.

**FIGURE 2:**
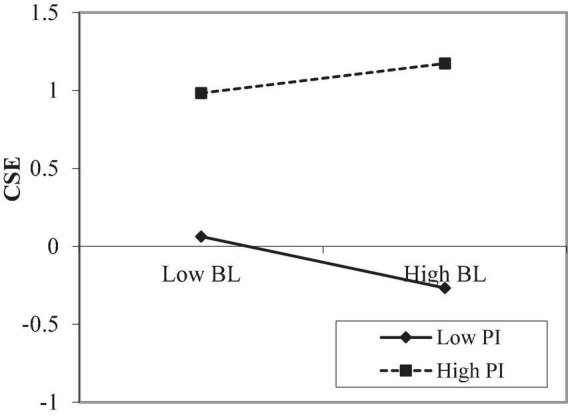
Moderating effect of PI. BL, benevolent leadership; CSE, creative self-efficacy; PI, professional identity.

**FIGURE 3:**
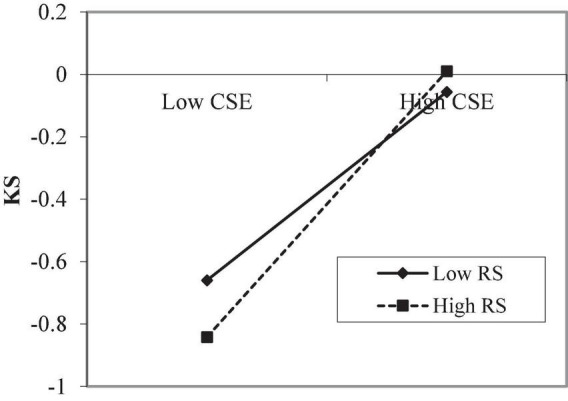
Moderating effect of RS. CSE, creative self-efficacy; KS, knowledge sharing; RS, research stress.

Process Macro Model 1 (Hayes, 2013, 2017) was used for moderating effect analysis. It can be seen from Table 4, when professional identity is a moderating variable, the interaction coefficient between the benevolent leadership and creative self-efficacy is significant (*Model 4*, *B* = 0.130, *SE* = 0.023, *p* < 0.001), it shows that professional identity plays a moderating role. Therefore, hypothesis 3 has been verified. In order to better understand the moderating effect of professional identity between the benevolent leadership and creative self-efficacy, the professional identity is taken at three different levels according to the average value and the average value plus or minus a standard deviation (M − 1 SD, M, M + 1 SD). When the level of professional identity is low (M − 1 SD), creative self-efficacy will increase by −0.164 standard deviations for every 1 standard deviation increase in benevolent leadership. When the level of professional identity is high (M + 1 SD), creative self-efficacy will increase by 0.095 standard deviation for every 1 standard deviation increase in benevolent leadership (Table 5). This suggests that the lower the professional identity, the stronger the negative influence of benevolent leadership on creative self-efficacy and the higher the professional identity, the stronger the positive influence of benevolent leadership on creative self-efficacy. The simple slope analysis diagram of benevolent leadership and creative self-efficacy drawn with professional identity as the moderating variable reflects the same rule (Figure 2). It can be seen from Table 4, when research stress is a moderating variable, the interaction coefficient between the creative self-efficacy and research stress is significant (*Model 5*, *B* = 0.062, *SE* = 0.014, *p* < 0.01), it shows that research stress plays a moderating role. Therefore, hypothesis 4 has been verified. In order to better understand the moderating effect of research stress between the creative self-efficacy and knowledge sharing, the research stress is taken at three different levels according to the average value and the average value plus or minus a standard deviation (M − 1 SD, M, M + 1 SD). When the level of research stress is low (M − 1 SD), knowledge sharing will increase by 0.302 standard deviations for every 1 standard deviation increase in creative self-efficacy. When the level of research stress is high (M + 1 SD), knowledge sharing will increase by 0.427 standard deviation for every 1 standard deviation increase in creative self-efficacy (Table 5). This suggests that when the research stress level is high, the correlation between creative self-efficacy and knowledge sharing is closer. The simple slope analysis diagram of creative self-efficacy and knowledge sharing drawn with research stress as the moderating variable reflects the same rule (Figure 3).

**TABLE 5:** Moderating effect on different moderated levels of PI and RS.

	Effect	SE	*t*	*P*	LLCI	ULCI
PI (M - 1 SD)	–0.164	0.035	–4.665	0.000	–0.233	–0.095
PI (M)	–0.035	0.027	–1.300	0.194	–0.087	0.018
PI (M + 1 SD)	0.095	0.032	2.995	0.003	0.033	0.157
RS (M - 1 SD)	0.302	0.035	8.522	0.000	0.232	0.372
RS (M)	0.364	0.029	12.589	0.000	0.308	0.421
RS (M + 1 SD)	0.427	0.039	10.944	0.000	0.350	0.503

PI, professional identity; RS, research stress; M, mean; SD, standard deviation.

**FIGURE 4:**
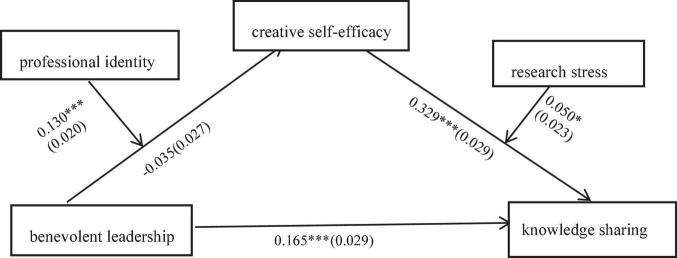
A moderated mediating effect model results. *P < 0.05, ***P < 0.001; the coefficients in the model are unstandardized, with standard errors in parentheses.

In order to better explore the relationship between the five variables, in SPSS, Process Macro Model 21 (Hayes, 2013, 2017) was used for moderated mediating effect analysis, the results are shown in Figure 4. According to Table 6, both professional identity and research stress moderated the mediating effect of creative self-efficacy, but the direction and magnitude of the moderating effect were different. When professional identity and research stress were positive one standard deviation (M + 1 SD), the indirect effect value was 0.036. When professional identity was negative one standard deviation (M − 1 SD) and research stress was positive one standard deviation (M + 1 SD), the indirect effect value was −0.062. This suggests that when the professional identity level was high and the research stress level was high, the indirect effect was the positive stronger; when the professional identity level was low and the research stress level was high, the indirect effect was negative stronger.

**TABLE 6:** Mediating effects of CSE on different moderated levels of PI and AS.

PI	RS	Effect	BootSE	BootLLCI	BootULCI
M-1 SD	M-1 SD	–0.046	0.013	–0.074	–0.024
M-1 SD	M	–0.054	0.014	–0.083	–0.028
M-1 SD	M + 1 SD	–0.062	0.017	–0.098	–0.031
M	M-1 SD	–0.010	0.008	–0.026	0.006
M	M	–0.011	0.009	–0.030	0.006
M	M + 1 SD	–0.013	0.011	–0.036	0.007
M + 1 SD	M-1 SD	0.026	0.010	0.010	0.049
M + 1 SD	M	0.031	0.010	0.012	0.054
M + 1 SD	M + 1 SD	0.036	0.012	0.014	0.062

CSE, creative self-efficacy; PI, professional identity; RS, research stress; M, mean; SD, standard deviation.

## Discussion

This study took 1,083 postgraduate supervisors as participants to explore the relationships among benevolent leadership, knowledge sharing, creative self-efficacy, professional identity and research stress, and built a moderated mediating effect model. This study explores the mechanism and boundary conditions of benevolent leadership’s influence on knowledge sharing, and answers not only “how” benevolent leadership influences knowledge sharing, but also “when” the influence is stronger or weaker. The results of this study enrich the literature and theory of benevolent leadership and knowledge sharing, and also have some implications for management practice.

### Theoretical implications

First, deepen the understanding of knowledge sharing of postgraduate supervisors and enrich relevant research. In China, postgraduate supervisors are mainly responsible for two tasks: cultivate postgraduates and conducting scientific research (Lu, 2017). Although the post of postgraduate supervisor belongs to academic position, the practitioners of academic position who do not have the qualification of postgraduate supervisor cannot cultivate postgraduates (Yin et al., 2021). Postgraduate supervisors have a closer relationship with knowledge sharing than those in other academic positions. (1) Cultivating postgraduates requires postgraduate supervisors to impart and share their knowledge with postgraduate (Cheng and Cao, 2021). (2) Postgraduate supervisors have accumulated certain knowledge during scientific research, and they have the ability and quality to share knowledge (Sun et al., 2019). (3) Knowledge sharing can help postgraduate supervisors obtain more information and resources, which benefits both individuals and organizations. Therefore, they play an important role in the academic development of an organization and it is particularly necessary to study the knowledge sharing of postgraduate supervisors. In the previous empirical studies, there are few studies that take postgraduate supervisor as participants, and even fewer studies that explore how leadership style influences the psychology and behavior of postgraduate supervisor. This study proves that benevolent leadership can positively promote the creative self-efficacy and knowledge sharing of postgraduate supervisor, and explores the influence of professional identity and research stress, which deepens the understanding of knowledge sharing and enriches the relevant research on postgraduate supervisor.

Second, enriches the theoretical research of benevolent leadership. Different cultural backgrounds give birth to different leadership theories. Benevolent leadership is rooted in the thought of “benevolence” in traditional Chinese culture (Farh and Cheng, 2000). “Benevolence” emphasizes friendship, help, understanding, sympathy, and tolerance among people. In terms of leadership behavior, leaders should not only be responsible for the development of the organization and the completion of work tasks, but also care for the personal well-being and living conditions of their subordinates (Cheng et al., 2004). This kind of care will not only extend to the subordinates’ families and matters outside their work, but also show their understanding of difficulties encountered in subordinates’ work and tolerance of mistakes made by subordinates. Benevolent leadership emphasizes that the leaders’ personalized, comprehensive and persistent care for the well-being of their subordinates is highly consistent with Chinese values, behavior patterns, and interpersonal communication models. It has been widely welcomed by Chinese and widely exists in Chinese organizations around the world (Lin et al., 2018), which has promoted the development and performance improvement of the organizations (Wang et al., 2018). However, compared with other types of leadership styles, there is still less research on benevolent leadership. This study constructs a moderated mediating effect model with benevolent leadership as the independent variable, knowledge sharing as the dependent variable, creative self-efficacy as the mediating variable, and professional identity and research stress as the moderating variables. It has accumulated the relevant literature of benevolent leadership research and enriched the theoretical research related to benevolent leadership.

Third, discusses the influence of benevolent leadership on knowledge sharing. From the social exchange theory, it can be seen that individuals always tend to follow the principle of reciprocity for equal value social exchange (Blau, 1964). Although individuals always tend to hide knowledge rather than share it in order to win respect and prestige (Park et al., 2017; Wang et al., 2020), caring and supportive behaviors of benevolent leaders inspire gratitude among subordinates. When leaders show sympathy and concern for subordinates, postgraduate supervisor will also give rewards with corresponding positive behaviors (Li et al., 2016). This study confirms the positive effect of benevolent leadership on knowledge sharing, highlights the important effect of benevolent leadership on knowledge sharing, and deepens the understanding of the relationship between benevolent leadership and knowledge sharing.

Fourth, explored the mediating effect of creative self-efficacy. After confirming the positive influence of benevolent leadership on knowledge sharing, it is particularly necessary to explore the internal mechanism of this influence. This study proves that creative self-efficacy has a partial mediating effect between benevolent leadership and knowledge sharing, that is, benevolent leadership can not only directly affect knowledge sharing, but also indirectly affect knowledge sharing through creative self-efficacy. Since the improvement of the creative self-efficacy of postgraduate supervisor is what benevolent leaders hope to see, social exchange theory can explain the positive role of benevolent leaders in creative self-efficacy (Yang et al., 2011). Social cognitive theory can also explain the positive effect of benevolent leadership on creative self-efficacy. Since social cognitive theory holds that social persuasion is beneficial to improve individual self-efficacy, trust, encouragement, and praise from leaders, as an important kind of social persuasion, are conducive to improving postgraduate supervisors’ creative self-efficacy (Zhang et al., 2018). Social cognitive theory emphasizes that self-efficacy is the key factor leading to individual behavior (Bandura, 1977). Therefore, the higher the creative self-efficacy of postgraduate supervisors, the more confidence in themselves, the more likely to appear knowledge sharing (Bandura, 1982). From the above discussion, it can be seen that the creative self-efficacy plays a mediating role, which not only reflects the relationship between benevolent leadership and creative self-efficacy but also reflects the relationship between creative self-efficacy and knowledge sharing. In a word, benevolent leadership increases knowledge sharing by improving the creative self-efficacy of postgraduate supervisors.

Fifth, tested the moderating effect of professional identity and research stress. Previous studies mainly used professional identity as an independent variable, dependent variable and mediator variable, and rarely used it as a moderator variable. This study believes that professional identity reflects an individual’s attitude toward his occupation, and different attitudes will bring different effects on the relationship between variables (Zhao et al., 2022). Therefore, professional identity can also be used as a moderating variable in the study. The results show that the relationship between benevolent leadership and creative self-efficacy is different under different levels of professional identity. When the level of professional identity is high (M + 1 SD), the moderating effect of professional identity is significant. With the increase of benevolent leadership, the level of creative self-efficacy is also increasing. When the level of professional identity is general (M), the moderating effect of professional identity is not significant. When the level of professional identity is low (M − 1 SD), the moderating effect of professional identity is significant. With the increase of benevolent leadership, the creative self-efficacy decreases. The emergence of this situation, on the one hand, makes us deepen our understanding of the importance of professional identity, and on the other hand, illustrates once again that the effectiveness of leadership behavior is different under different conditions (Zhang et al., 2018). When subordinates do not agree with their profession, the caring and tolerance to mistakes of benevolent leadership may make subordinates lower their requirements on themselves, and thus make them more mediocrity and muddle along in work, and even produce work withdrawal behavior or workplace deviant behavior (Ding et al., 2022).

The research stress is an important environmental variable affecting the work and life of postgraduate supervisor. According to the challenge-hindrance stressors model, research stress belongs to challenge stressor, which brings more gains and growth to employees (Cavanaugh et al., 2000). Under different research stress conditions, the relationship between creative self-efficacy and knowledge sharing is different. The higher the research stress, the closer the relationship between creative self-efficacy and knowledge sharing will be. According to the theory of emotional arousal, higher research stress will stimulate the passion and vitality of postgraduate supervisor with high creative self-efficacy (Bunce and West, 1994), they will be more confident, have more positive behaviors, and therefore have more knowledge sharing behaviors. The results of this study not only prove the moderating effect of research stress on creative self-efficacy and knowledge sharing, but also prove the positive effect of challenge stress on work.

Sixth, deepen the understanding of the effectiveness of leadership behavior. Whether a certain leadership behavior is effective or how the effect is, there are differences in different influence mechanisms and different situations (Farh and Cheng, 2000). Many studies have shown that the same leadership behavior is effective in one situation and may not be effective in another situation (Li et al., 2014; Zhang et al., 2018). This study fully considers the difference and complexity of the situation, takes professional identity and research stress as moderating variables, comprehensively considers the influence of the two moderating variables on the mediating effect. When the postgraduate supervisor has a high level of professional identity and high research stress, he has the will to pay for his own work from the individual perspective, and an atmosphere to promote work harder from the environmental perspective. Therefore, benevolent leadership has a positive influence on knowledge sharing through creative self-efficacy. When postgraduate supervisors have low professional identity and high research stress, they will not recognize their own work from the perspective of individuals and are even less willing to pay for their work. From the perspective of the environment, research stress has become a burden. Therefore, benevolent leadership has a negative influence on knowledge sharing through creative self-efficacy. The moderated mediating effect model constructed in this study comprehensively explains the influence of multiple moderating variables on the relationship between benevolent leadership and knowledge sharing, deepening the understanding of the effectiveness of leadership behavior.

### Practical implications

First, show benevolent leadership behavior and promote knowledge sharing. Postgraduate supervisors are typical knowledge workers, who have strong autonomy and creative in their work. The leadership style of caring, support and tolerance shown by benevolent leaders highly conforms to the characteristics of postgraduate supervisors’ work. In practical work, leaders can encourage postgraduate supervisors to share more knowledge and make contributions to the development of the organization with their own knowledge by caring about their work and life, solving their difficulties, creating a relaxed working environment, and other benevolent leadership behaviors (Li et al., 2022). Second, increase job opportunities and improve creative self-efficacy. According to the social cognitive theory, the improvement of self-efficacy is influenced by previous successful experience, verbal persuasion, and other factors (Bandura, 1982). Organizations can provide employees with more job opportunities and personal development opportunities through job enrichment, job expansion, participation in decision-making and encouraging voice behavior. By letting employees improve their personal quality and accumulate successful experience in their work, we can create conditions for improving their creative self-efficacy (Xia et al., 2022). On this basis, leaders can improve subordinates’ creative self-efficacy by creating a relaxed working environment and encouraging subordinates to make bold innovations in their work. Third, cultivate the spirit of respecting work and enhance professional identity. In the recruitment process, the organization should pay attention to the selection of employees who like to engage in the work, and let the employees with high compatibility of ability, temperament, personality, and occupation join the organization (Horvath et al., 2018). In work, it is necessary to increase the training of employees, so that employees fully realize the significance of their work. In the process of managing the career of employees, the development prospect of employees in the organization should be planned (Wang et al., 2017), so that employees can see the future and hope, so as to improve their professional identity. Fourth, according to the actual situation of the organization, appropriately increase research stress. Organizations can create certain stress scenarios according to their industries and their own development stages to increase the motivation and enthusiasm of employees (Zhang et al., 2018). When the organization carries out stress management, it must pay attention to the actual situation. If the stress is too small, it will not motivate the employees. If the pressure is too large, the employees will lose confidence and motivation (Tang et al., 2022). Fifth, considering different situational factors and take appropriate leadership behavior. Situational factors are important factors affecting the effectiveness of leadership behavior (Li et al., 2015). Due to the complexity of the situation, there may be a variety of situational factors affecting leadership behavior (Li et al., 2022). It is necessary to fully consider the influence of different situational combinations on the effectiveness of leadership behavior and adopt different leadership behaviors according to different situations.

### Limitations and future directions

There are still some shortcomings in this study, which need to be further improved in subsequent studies. First, this manuscript is a cross-sectional study at a single time point, and all the data are from the participants themselves. Although there is no serious common method bias problem, it is not conducive to infer the causal relationship of variables. In future studies, data collection from multiple sources, longitudinal tracking surveys, and experimental methods should be attempted to further verify the relationship between variables. Second, due to the limitation of conditions, this manuscript adopts the snowball method to collect data by using the individual’s social relationship network, so the generalizability and representativeness of the research results deserve further analysis. In the future, the sampling range should be enlarged to make the results more robust. Third, this manuscript only discusses the linear relationship between variables, but does not consider the non-linear relationship between variables. Pierce and Aguinis (2013) once proposed the “Too much of a good thing” effect in the field of management, that is, there may be a non-linear inverted “U–shaped” relationship between variables. Future researches can explore the curvilinear relationships between variables. Fourth, although this paper discusses the mechanism and boundary conditions of benevolent leadership’s influence on knowledge sharing, the process of leadership’s influence on behavior is very complex, and there are still some other variables that can mediate or moderate the relationship between them (Wang and Cheng, 2010). In the future, we should try to explore other mediating variables (e.g., organizational commitment, job satisfaction, psychological contract, perceived insider status) and moderating variables (e.g., gender, education, traditionality, perceived climate of team Cha-xu) to enrich the relevant research on benevolent leadership.

## Conclusion

This study constructs a moderated mediating effect model with benevolent leadership as the independent variable, knowledge sharing as the dependent variable, creative self-efficacy as the mediating variable, and professional identity and research stress as the moderating variables. The results showed that benevolent leadership positively influence knowledge sharing, creative self-efficacy partially mediated the relationship between benevolent leadership and knowledge sharing, professional identity moderated the relationship between benevolent leadership and creative self-efficacy, and research stress moderated the relationship between creative self-efficacy and knowledge sharing. Professional identity and research stress jointly moderate the mediating effect of creative self-efficacy between benevolent leadership and knowledge sharing.

## Data availability statement

The original contributions presented in this study are included in this article/supplementary material, further inquiries can be directed to the corresponding author.

## Ethics statement

The studies involving human participants were reviewed and approved by the Ethics Committee of Institute of Psychology and Behavior of Henan University. All participants agreed to participate in the study. The patients/participants provided their written informed consent to participate in this study.

## Author contributions

Both authors were involved in developing, editing, reviewing, and providing feedback for this manuscript and approved the final version to be published.

## Appendix

### Benevolent leadership

The leader usually give me his (her) assiduous and thoughtful attention.

The leader will care about my personal life.

The leader’s care for me will extend to my family.

The leader will give careful and considerate care to the subordinates who have been together for a long time.

When I meet with difficulties, the leader will give me help in time.

### Knowledge sharing

I am willing to share my knowledge and experience with others.

When participating in the discussion, I will provide my own opinions as much as possible.

I will try my best to answer questions raised by my colleagues.

When my colleague needs help, I will try my best to provide him with the information and documents he needs.

I think sharing knowledge and experience with others is a very fulfilling thing.

### Creative self-efficacy

I think I’m good at putting forward new ideas.

I have confidence in my ability to solve problems creatively.

I have tips to further supplement and improve others’ views.

I am good at finding new ways to solve problems.

### Professional identity

As a postgraduate supervisors, I often feel respected.

I am proud to be a postgraduate supervisors.

I feel insulted when someone makes groundless accusations against the postgraduate supervisor group.

As a postgraduate supervisor, I can realize my value.

I will be very happy when I see or hear the words praising the postgraduate supervisor.

I would like to mention that I am a postgraduate supervisor.

### Research stress

I am worried about how to complete the research task.

I feel a lot of stress from my research work.

I feel depressed and unhappy because of my research work.
